# A review on associated factors and management measures for sarcopenia in type 2 diabetes mellitus

**DOI:** 10.1097/MD.0000000000037666

**Published:** 2024-04-19

**Authors:** Yi Zhang, Kemeng Zhang, Sui Huang, Wenhan Li, Ping He

**Affiliations:** a Department of Geriatrics, Union Hospital, Tongji Medical College, Huazhong University of Science and Technology, Wuhan, China.

**Keywords:** muscle strength, sarcopenia, skeletal muscle mass, type 2 diabetes mellitus

## Abstract

Type 2 diabetes mellitus (T2DM) is a chronic metabolic disease characterized by hyperglycemia, insulin resistance, and insufficient insulin secretion. Sarcopenia, as a new complication of diabetes, is characterized by the loss of muscle mass and the progressive decline of muscle strength and function in T2DM patients, which has a serious impact on the physical and mental health of patients. Insulin resistance, mitochondrial dysfunction, and chronic inflammation are common mechanisms of diabetes and sarcopenia. Reasonable exercise training, nutrition supplement, and drug intervention may improve the quality of life of patients with diabetes combined with sarcopenia. This article reviews the relevant factors and management measures of sarcopenia in T2DM patients, in order to achieve early detection, diagnosis, and intervention.

## 1. Introduction

Diabetes is a common chronic disease, and the incidence of type 2 diabetes mellitus (T2DM) is rising all over the world.^[1]^ According to previous research, the number of people with diabetes has quadrupled in the past 30 years worldwide, and about 1 in 10 adults in the world has diabetes, of which 90% have T2DM.^[2]^ Anagnostis et al^[3]^ showed that patients with T2DM had an increased risk of sarcopenia. In addition to microvascular and macrovascular complications, sarcopenia has been described as a new complication observed in the elderly population with T2DM.^[4]^

The definition and diagnosis of sarcopenia has been continuously developing. In 2010 and 2014, the European Working Group on Sarcopenia (EWGOS 1) and the Asian Working Group on Sarcopenia (AWGS 1) agreed that sarcopenia is a syndrome characterized by progressive and generalized loss of skeletal muscle mass and strength.^[5,6]^ In 2019, the AWGS updated the threshold value of the sarcopenia criteria (AWGS 2). The EWGOS definition was also updated (EWGOS 2). In EWGOS 2, sarcopenia is defined as an age-related degenerative skeletal muscle disease, which is characterized by progressive and systemic decline in muscle mass, muscle strength, and function. Sarcopenia is probable when low muscle strength is detected. A sarcopenia diagnosis is confirmed by the presence of low muscle quantity or quality. When low muscle strength, low muscle quantity/quality, and low physical performance are all detected, sarcopenia is considered severe.^[7]^

Sarcopenia is a characteristic of other syndromes associated with muscle atrophy, such as cachexia, sarcopenia obesity, and frailty.^[5]^ Low muscle mass is a typical phenotype of sarcopenia, while cachexia is characterized not only by low muscle mass but also by weight loss and anorexia.^[8]^ A loss in lean body mass is accompanied by excessive accumulation of adipose tissue. This state is called sarcopenic obesity. Coexistence of obesity and sarcopenia can lead to adverse health consequences.^[9]^ Several studies have shown that the mechanism of sarcopenia obesity is related to chronic inflammation.^[10,11]^ There is evidence that sarcopenia with obesity may be associated with higher levels of metabolic disorders and an increased risk of mortality than obesity or sarcopenia alone.^[12]^ Sarcopenia is closely related to frailty, and is the main component of frailty syndrome. Sarcopenia and frailty are considered to be powerful predictors of disability and death in the elderly.^[13]^

The muscle mass and strength of T2DM patients with sarcopenia decreased significantly, which greatly increased the risk of falls, fractures,^[14]^ bed rest, and death^[15,16]^ in the elderly, seriously affected the quality of life of patients, and increased the burden on families and society. Multiple interventions, including adequate nutrition, exercise training, good blood glucose control, and the use of appropriate hypoglycemic drugs, may help to delay and prevent the progress of disability.^[17]^ Therefore, it is necessary to be alert to the occurrence of sarcopenia in T2DM patients.

For clinician, it is particularly important to preliminarily understand the associated factors and management measures of T2DM with sarcopenia, which will help us better understand the relationship between T2DM and sarcopenia and other diseases, so as to better prevent and manage T2DM and sarcopenia, and improve the quality of life of patients. This article mainly reviews the relevant mechanisms, associated factors, and management measures of T2DM with sarcopenia, providing a basis for peers to understand and manage T2DM patients with sarcopenia.

## 2. Mechanism of T2DM with sarcopenia

The common mechanisms of T2DM and sarcopenia mainly include insulin resistance, mitochondrial dysfunction, systemic chronic inflammation, fat accumulation, etc. In T2DM patients, hyperglycemia toxicity and increased fatty acid concentration will lead to insulin secretion and insulin resistance, which in turn will promote the rise of blood sugar and fatty acids. The decrease of peroxisome-proliferator–activated receptor γ coactivator 1 in patients with T2DM suggests that mitochondrial dysfunction may lead to cellular lipid accumulation and insulin resistance,^[18]^ while inflammatory factors released from adipose tissue, such as tumor necrosis factor and interleukin-6, also has adverse effects on the insulin signaling pathway.^[19]^ The decrease in muscle mass is closely related to metabolic disorders, including decreased insulin sensitivity, impaired oxidative defense, and decreased mitochondrial function.^[20]^ The increase in levels of inflammatory markers is related to sarcopenia.^[21]^ Fat accumulation can reduce muscle quality and function,^[5]^ and promote insulin resistance and diabetes.^[22]^ Serum growth hormone, insulin-like factor-1 (IGF-1), and mechanical growth factor levels are lower in patients with sarcopenia,^[23]^ while the growth hormone/IGF-1 axis has a strong effect on protein, fat, and glucose metabolism. IGF-1 dysfunction can lead to T2DM.^[24]^

In addition, autophagy is closely related to the occurrence of T2DM and sarcopenia. The enhancement of autophagy can increase insulin sensitivity, reduce oxidative stress response, and thus regulate overall metabolism. Autophagy plays a potential role in boosting β-cell survival and preventing diabetes by suppressing ER stress, oxidative stress, and inflammation-induced injury in β-cells.^[25]^ A study found that mice with the key autophagy gene Atg7 knocked out experienced severe muscle atrophy and age-dependent strength decline, and autophagy inhibition exacerbated muscle loss during denervation and fasting.^[26]^ FoxO/4E-BP signal transduction may prevent skeletal muscle aging through autophagy. Boosting basal autophagy protects from age-related muscle dysfunction by promoting the selective degradation of misfolded proteins and dysfunctional organelles.^[27]^

## 3. Influencing factors related to sarcopenia in patients with T2DM

### 3.1. Age

Muscle health declines with age, which makes sarcopenia be more common in elderly diabetes. Most studies have shown that patients with T2DM and sarcopenia are older than patients without sarcopenia. Formiga et al^[28]^ showed that older age was associated with a higher prevalence of sarcopenia in elderly diabetes patients. Chen et al^[29]^ showed that the incidence of sarcopenia in patients with T2DM increased significantly with age, from 6.51% in the 60 to 69-year-old group to 27.67% in the 80-year-old group (*P* < .001). This is consistent with the research results of Fukuoka et al,^[30]^ Mori et al,^[31]^ and others.

### 3.2. Gender

The prevalence of sarcopenia in patients with T2DM is also related to individual gender, but the conclusions drawn from different studies are different. Some studies have shown a higher prevalence of sarcopenia in man,^[32–35]^ while others have shown a higher prevalence of sarcopenia in woman.^[29,36,37]^

In addition, in the study of de Freitas et al,^[33]^ it was found that according to EWGSOP1, the prevalence of sarcopenia group was 16.9%, most of which are men. When defined according to EWGSOP2, the prevalence of sarcopenia group was 7%, most of which are women (88%), and the prevalence of sarcopenia more than doubled compared with EWGSOP1 (16.9%) and EWGSOP2 (7%). The authors believe that the difference in prevalence is due to the modification of muscle mass and muscle strength standards.

### 3.3. Body mass index

Some studies have found that among patients with T2DM, patients with sarcopenia have significantly lower body mass index (BMI).^[30,38]^ A study of 1137 patients^[31]^ found that in Japanese patients with T2DM, the best cutoff point for BMI level with sarcopenia is 24.4 kg/m^2^ (area under the curve = 0.729, 95% confidence interval = 0.688–0.770, sensitivity = 0.587, specificity = 0.789). BMI <24 kg/m^2^ will increase the risk of sarcopenia. And the research^[39]^ also showed that BMI may be a practical index for screening sarcopenia, and can be used as a substitute index of gait speed in the diagnosis of sarcopenia to a certain extent.

### 3.4. Glycemic control

In some studies, glycosylated hemoglobin (HbA1c) is usually measured to evaluate the relationship between glycemic control and sarcopenia in T2DM patients. Some studies have shown that high levels of HbA1c increase the risk of sarcopenia in T2DM patients.^[34,40,41]^ Nakanishi et al^[42]^ showed that the HbA1c level of patients with sarcopenia was higher than that of patients without sarcopenia. Liny et al^[43]^ reached the same conclusion as Nakanishi et al.^[42]^ Sugimoto et al^[44]^ reported that the frequency of sarcopenia increased linearly with the level of HbA1c, especially in the lean population (HbA1c <6.5%: 7.0%; HbA1c ≥6.5% and <7.0%: 18.5%; HbA1c ≥7.0% and <8.0%: 20.3%; HbA1c ≥8.0%: 26.7%).

### 3.5. Diabetic complications

#### 3.5.1. Diabetic nephropathy.

Feng et al^[34]^ showed that diabetic nephropathy is a risk factor for sarcopenia in patients with diabetes. Yang et al^[45]^ reported that the incidence rate of sarcopenia in patients with T2DM is related to the decline of renal function. Çeliker et al^[46]^ also concluded that the incidence of sarcopenia in patients with diabetes is higher. Ida et al^[47]^ also concluded that the occurrence of sarcopenia in diabetes patients is significantly related to the reduction of urinary albumin level, urinary protein level, and glomerular filtration rate. However, Formiga et al^[28]^ have found that sarcopenia is not significantly increased in patients with more advanced chronic kidney disease.

#### 3.5.2. Diabetic neuropathy.

As for diabetic neuropathy, Yasemin et al^[48]^ pointed out that the incidence of sarcopenia in diabetic neuropathy patients was significantly higher than that in nondiabetic neuropathy patients (24.7%–8.9%). Similarly, in a study of 230 patients with T2DM using the Michigan neuropathy screening instrument questionnaire and physical examination to assess diabetic peripheral neuropathy (DPN), Oh et al^[49]^ reported that men with DPN had significantly lower grip strength than men without DPN; there was no significant correlation between grip strength and female neuropathy.

#### 3.5.3. Cardiovascular disease.

Visceral fat or muscle mass is determined to be associated with cardiac metabolic disease, especially in T2DM patients. Park et al^[50]^ showed that low appendiceal skeletal muscle mass and low grip strength are closely related to cardiovascular disease and coronary heart disease in T2DM. Liu et al^[51]^ reported that skeletal muscle mass and visceral fat area ratio were negatively correlated with 10-year cardiovascular disease risk in T2DM patients, especially in patients with high HbA1c levels.

### 3.6. Nonalcoholic fatty liver disease

Some studies have shown that sarcopenia is independently associated with the prevalence of nonalcoholic fatty liver disease (NAFLD) in patients with T2DM. For example, Seo et al^[52]^ reported that in patients with T2DM, the prevalence of sarcopenia in NAFLD patients was significantly higher than that in non-NAFLD patients, whether male or female: 45.5% versus 24.7% in men, respectively (*P* < .001); female 36.4% versus 23.9% (*P* < .001). In addition, in a study of 309 T2DM individuals who used fibrosis-4 index to evaluate the load of liver fibrosis, Sung et al^[38]^ reported that the prevalence of sarcopenia in T2DM patients was independently related to the load of liver fibrosis.

### 3.7. Cognitive impairment

Most studies show that sarcopenia is related to cognitive impairment, but this study is less in the diabetes population. A study on the elderly population in Japan shows that among diabetes patients, sarcopenia is positively correlated with mild cognitive impairment (MCI).^[36]^ Another study showed that in T2DM patients, compared to the non-MCI group, the MCI group had lower grip strength, walking speed, and bone mass index, and a higher incidence of sarcopenia (13% vs. 4%). This study also clarified that slow walking speed is the sole determinant criteria of sarcopenia of MCI in patients with type 2 diabetes mellitus.^[53]^ A cohort study showed that in T2DM patients, BIA measure of muscle mass loss over time was independently associated with cognitive decline globally and in the domains of memory and visuo-spatial/construction.^[54]^

## 4. Intervention measures

### 4.1. Physical activity

Research has shown that resistance training can improve muscle mass and prevent the occurrence of sarcopenia.^[55]^ A meta-analysis was conducted to investigate the effects of different exercise modes on muscle mass and physical performance in elderly patients with sarcopenia, indicating that muscle strength and gait speed can be improved through resistance training and mixed training (resistance training combined with other exercises such as balance, endurance, and aerobic training), but cannot be improved through whole-body vibration training.^[56]^ Hurst et al^[57]^ designed a suitable resistance training program, suggesting exercising twice a week, combined with upper and lower body exercises, with relatively high effort for 1 to 3 groups, repeated 6 to 12 times.

The American Diabetes Association pointed out that T2DM patients should accumulate at least 150 minutes of moderate intensity physical activity (40%–60% peak oxygen uptake or 75 minutes of high-intensity physical activity [60%–85% peak oxygen uptake]) every week to maintain or improve health.^[58]^ Aerobic exercise or resistance exercise can effectively improve the score and prevalence of metabolic syndrome in T2DM patients,^[59]^ HbA1c level,^[60]^ and endothelial function.^[61]^ In addition, joint training exceeding 150 minutes per week can more effectively reduce HbA1c levels.^[62]^ Some studies have shown that combined exercise (resistance-aerobic) serum kinesin cannot reverse type 2 diabetes peripheral neuropathy, but can delay the progress of the disease.^[63,64]^ Exercise may positively affect pathological factors related to neuropathy by promoting microvascular dilation, reducing oxidative stress, and increasing neurotrophic factors.^[64]^ Some studies have pointed out that high-intensity interval training may be an effective tool to improve the long-term vascular complications of T2DM.^[65]^ Therefore, exercise training has a positive effect on sarcopenia, T2DM, and its complications. Further research is needed to develop a reasonable and effective training plan.

### 4.2. Nutrition

In nonpharmacological interventions, excluding exercise, nutrition is another cornerstone that we need to pay attention to. Research shows that the reduction of energy intake in elderly patients with diabetes may lead to the reduction of BMI, which may be related to the increased risk of weakness and death.^[66]^ The European Society of Clinical Nutrition and Metabolism recommends guidelines for clinical nutrition and fluid replacement in geriatrics, with an energy intake of approximately 30 kcal/kg/d for the elderly,^[67]^ but these values need to be adjusted separately based on nutritional status, physical activity levels, disease status, and tolerance.

Dietary protein supplementation can increase muscle mass^[68]^ and control blood sugar and lipid levels, which helps elderly patients with diabetes achieve better metabolic control.^[69]^ One study suggested that the minimum daily protein intake of elderly patients with diabetes was 0.8 g/kg/d to maintain good metabolic control; for elderly T2DM patients with chronic kidney disease stages 1 to 3, the appropriate protein intake is about 0.8 to 1.0 g/kg/d.^[70]^ Therefore, it is recommended to supplement adequate protein for type 2 diabetes patients with sarcopenia, except for patients with end-stage renal failure.

The intake of whey protein, essential amino acids, and vitamin D helps improve muscle mass and physical performance.^[71]^ Some studies have shown that low vitamin D level will increase the risk of type 2 diabetes in the elderly,^[72]^ but in the randomized controlled study on vitamin D, it has not been found that vitamin D supplementation can effectively improve the blood sugar level of type 2 diabetes patients.^[73]^

Polyunsaturated fatty acids (PUFAs) have attracted great attention, including omega-3 fatty acids, α-Linoleic acid, eicosapentaenoic acid, docosapentaenoic acid, and docosahexaenoic acid. Multiple studies have shown that supplementing omega-3 fatty acids, eicosapentaenoic acid, and docosahexaenoic acid can reduce inflammation levels in the population.^[74,75]^ A review pointed out that supplementation of 0.42 to 5.2 g PUFAs every day for at least 8 weeks may become an alternative therapy for type 2 diabetes, especially in Asian subjects.^[76]^ Of course, PUFAs can also serve as alternative therapies for sarcopenia.^[77]^ Further research is needed on the specific dosage, frequency, and duration of supplementation.

The Mediterranean diet is currently a widely studied dietary approach that includes beans, nuts, fish and olive oil, wine, as well as moderate intake of meat and dairy products. Research has confirmed that a Mediterranean diet is beneficial for cardiovascular risk factors, blood sugar control, and BMI.^[78]^ Compared with ketogenic diet, the Mediterranean diet pattern has better sustainability for patients with type 2 diabetes.^[79]^ At present, the Mediterranean diet is beneficial for frailty, functional impairment,^[80]^ and cognitive function,^[81]^ but there is no evidence to suggest a positive effect on sarcopenia.^[80,82]^ Whether the Mediterranean diet pattern is beneficial to type 2 diabetes patients with sarcopenia needs further research.

### 4.3. Hypoglycemic drugs

#### 4.3.1. Metformin.

At present, the impact of metformin on the population with sarcopenia is not clear, and more clinical research evidence is needed. Among a group of 1732 T2DM patients,^[29]^ participants who took metformin alone or in combination with other medications had a lower risk of developing sarcopenia compared to those who did not take the medication. Cui et al^[35]^ also reached a consistent conclusion. Metformin may regulate myostatin in mouse skeletal muscle cells through the AMPK-FoxO3a-HDAC6 axis, thereby damaging muscle function.^[83]^ A review suggests that the use of metformin can lead to muscle wasting in elderly patients who do not exercise adequately.^[84]^

#### 4.3.2. Sulfonylureas.

Sulfonylurea drugs may cause muscle atrophy. A search of atrophy-related signaling in the Food and Drug Administration Adverse Event Reporting System showed that muscle atrophy occurred in 0.27% of human subjects who had used glibenclamide, whereas no atrophy was reported in subjects who had used sulfonylureas 9 or glinides.^[85]^ This study suggests that drug-induced atrophy is achieved through KATP channel blockade and enhanced mitochondrial SDH activity. Ishii et al^[86]^ found that no changes in skeletal muscle mass were reported in patients with T2DM assigned to sulfonylureas.

#### 4.3.3. Thiazolidinediones.

The effect of thiazolidinediones on muscle atrophy is not yet clear. An early multicenter longitudinal study by Lee et al^[87]^ reported that the percentage loss in total or appendicular lean mass in men with diabetes treated with thiazolidinediones was significantly less than that in normoglycemic men. This result cannot be explained by differences in blood sugar levels. The mechanism that led to these findings is still uncertain, possibly through upregulation of peroxisome-proliferator–activated receptor-γ coactivator 1-α gene expression enhances fatty acid oxidation, thereby converting glycolytic fibers into antioxidant fatigue fibers.

In another study, patients randomized to rosiglitazone had no significant changes in lean mass and had more subcutaneous abdominal adipose tissue area.^[88,89]^

Shea et al^[90]^ examined the influence of pioglitazone and resistance training on body composition in older (65–79 years) nondiabetic overweight/obese men and women during weight loss. This trial performed in individuals without T2DM has shown that men who were given pioglitazone lost more thigh muscle volume, compared to men who were not given pioglitazone. However, muscle resistance training was effective in counteracting muscle loss.

#### 4.3.4. Dipeptidyl peptidase-4 inhibitors.

DDP4-I may reduce muscle mass loss. Rizzo et al^[91]^ pointed out that compared with sulfonylurea, the use of dipeptidyl peptidase-4 inhibitors (DPP4-i) may have a beneficial effect on the prevention of muscle mass loss and its function. Sencan et al^[92]^ also obtained consistent results. In addition, it also showed that DPP4-i had a positive effect on blood glucose control. A retrospective observational study also found that DPP4-i attenuates the decline of skeletal muscle mass in patients with type 2 diabetes.^[93]^ DPP4 has potential as a potential treatment for sarcopenia.

#### 4.3.5. Sodium-glucose transport protein 2 inhibitors.

With regard to the study of SGLT2 inhibitors, Sasaki et al^[94]^ reported that luseogliflozin treatment can slightly reduce the skeletal muscle mass index (SMI), accompanied by a reduction in body fat. Similarly, the research of Kamei et al^[95]^ showed that after treatment with tofogliflozin, patients’ weight and HbA1c levels decreased significantly, as did skeletal muscle mass and SMI. Some studies have shown that skeletal muscle mass will not decrease^[96]^ or remain unchanged after treatment with SGLT2i.^[97]^

#### 4.3.6. Glucagon-like peptide-1 receptor agonists.

Li et al^[98]^ reported that following 12-week liraglutide treatment, body weight, waist circumference, total fat and lean mass, fat percentage were significantly reduced from baseline. Li et al^[98]^ also found significant correlations between weight loss and increases in both plasma atrial natriuretic peptide and brain natriuretic peptide levels. Similar results were found in another study.^[99,100]^ Conversely, another trial shows that none of the patients under liraglutide treatment has become sarcopenic. Unexpectedly, 5 patients showed even an improvement in SMI.^[101]^

#### 4.3.7. Insulin.

Tanaka K and colleagues suggested that reduction in endogenous insulin secretion is an independent risk factor of sarcopenia in men with T2DM.^[101]^ A retrospective observational study reported that insulin treatment significantly increased the 1-year change in SMI compared with noninsulin-treated group. This research data suggested that insulin treatment could attenuate the progression of sarcopenia in patients with type 2 diabetes.^[102]^ Sugimoto et al^[103]^ showed that correction of poor glycemic control and use of insulin were significantly associated with the increase in skeletal muscle mass or gait speed in Japanese patients with type 2 diabetes. During the follow-up period, the frequency of sarcopenia marginally increased, and the means of SMI, handgrip strength, and gait speed did not show any changes. However, on dividing into 5 groups depending on the degree of changes in HbA1c value, the patients with a decrease of ≥1% in HbA1c exhibited a significant increase in SMI. Their analysis revealed similar results for gait speed but not handgrip strength.^[103]^

### 4.4. Statin

It is well known that statins can reduce blood lipid levels, slow down the progression of atherosclerosis, and reduce the risk of cardiovascular disease. Some patients with T2DM often use statins. Previous studies have shown that statins promote muscle reduction, and their mechanisms may be related to a decrease in coenzyme Q10 and the ubiquinol-proteasome pathway.^[104,105]^ Some studies have shown that statins do not increase sarcopenia,^[106]^ and can even prevent and reduce the occurrence of sarcopenia.^[107,108]^ Statins can increase SMI in patients with heart disease.^[109,110]^ A study has shown that in older individuals undergoing progressive resistance training, combination therapy with metformin and statins can increase muscle fiber area, but has no significant effect on strength.^[111]^ The effect of statins on sarcopenia in patients with T2DM needs further research.

## 5. Conclusion

In summary, we conclude that aging, low BMI, high level of HbA1c, complications of diabetes, combination of NAFLD or CI may increase the risk of sarcopenia in T2DM. In addition, we also advocate that a combination of exercise, nutrition, and medication is more beneficial for managing T2DM patients with sarcopenia. Due to various factors such as regional differences, sample differences, and different experimental methods, some experimental conclusions may also contradict each other, which requires extensive research by scholars to confirm. In the future, some related factors are likely to become indicators for screening sarcopenia, and the diagnostic criteria for sarcopenia will be more detailed and reliable. Numerous studies have shown that the combination of exercise and nutrition, as well as the use of some hypoglycemic drugs, is beneficial for patients with T2DM complicated with sarcopenia. However, the specific details of exercise and nutrition strategies, such as frequency, time, and method, are still unclear, which may require further research. Drug and nondrug combination treatment is the normal state of T2DM with sarcopenia. We need more research to determine the interaction between drugs and between drugs and nondrugs, which may be the direction we need to work hard in the future.

## Author contributions

**Writing—original draft:** Yi Zhang.

**Writing—review & editing:** Kemeng Zhang, Sui Huang, Ping He.

**Supervision:** Wenhan Li.
